# Interprofessional collaboration between health sciences librarians and health professions faculty to implement a book club discussion for incoming students

**DOI:** 10.5195/jmla.2019.563

**Published:** 2019-07-01

**Authors:** Jen Haley, Rebecca Carlson McCall, Meg Zomorodi, Lisa de Saxe Zerdan, Beth Moreton, Lee Richardson

**Affiliations:** Graduate Student, School of Nursing, University of North Carolina at Chapel Hill, Chapel Hill, NC, jenhaley@email.unc.edu; Clinical Librarian, Health Sciences Library, University of North Carolina at Chapel Hill, Chapel Hill, NC, rcarlson@unc.edu; Assistant Provost and Director, Office of Interprofessional Education and Practice; Clinical Associate Professor, School of Nursing; and Josiah Macy Faculty Scholar and Well Care Home Health Faculty Scholar; University of North Carolina at Chapel Hill, Chapel Hill, NC, meg_zomorodi@unc.edu; Senior Associate Dean for Master’s in Social Work Education and Clinical Associate Professor, School of Social Work, University of North Carolina at Chapel Hill, Chapel Hill, NC, lzerden@email.unc.edu; Clinical Librarian, Health Sciences Library, University of North Carolina at Chapel Hill, Chapel Hill, NC, emoreton@email.unc.edu; Information Discovery and Metadata Librarian, Health Sciences Library, University of North Carolina at Chapel Hill, Chapel Hill, NC, richarlm@email.unc.edu

## Abstract

**Background:**

The following case example provides an overview of one innovative way to engage health professions faculty with health sciences librarians in the development of an interprofessional book discussion and identifies strategies to address implementation challenges. Academic health sciences librarians worked with the Interprofessional Education (IPE) Steering Committee to organize interprofessional book discussion groups for incoming health professions students. This inaugural book discussion brought together students and faculty of different disciplines to engage students in “learning from, with, and about” other professions.

**Case Presentation:**

*When Breath Becomes Air*, by Paul Kalanithi, allowed involved discussions on important health sciences issues. The project included outreach, designing pre- and post-surveys, scheduling participants, and communicating with all participants before, during, and after the event. A total of seventy-nine students and thirty-six faculty, representing all health professions schools, participated in the small group IPE book discussions over two weeks.

**Conclusions:**

Small group book discussions have been shown to be an effective tool to engage students and faculty in IPE. The results of the participant surveys were positive, and the IPE Steering Committee found value in including health sciences librarians throughout the process. Lessons learned from the pilot project include needing an efficient scheduling system, strongly communicating at all stages of the project, and starting the planning process months ahead of time. The IPE Steering Committee plans to conduct similar book discussions every fall semester moving forward and explore options for other IPE events.

## BACKGROUND

Interprofessional education (IPE) occurs when “students from two or more professions learn about, from and with” one another with the purpose of enabling effective collaboration to improve health outcomes [1], whether didactically in course work, in clinical learning environments [2], or both [3]. However, IPE activities require more than just bringing students from diverse disciplines together [4]. Careful considerations about professional cultures, histories, and perspectives are necessary to create robust IPE learning experiences that are meaningful to and appropriate for all learners [4]. In health professions that are frequently siloed, it can be a challenge to plan an event that carries meaning for all participants.

The literature has identified several barriers to implementation of IPE activities that can lead to didactic activities being “one and done” and conducted without evaluation metrics. In a systematic review of interprofessional education, ten implementation challenges were identified related to: (1) curriculum, (2) leadership, (3) resources, (4) stereotypes, (5) students’ diversity, (6) the concept of IPE, (7) teaching, (8) enthusiasm, (9) professional jargons, and (10) accreditation [5]. In particular, the difficulty of integrating IPE into an already packed curriculum, reconciliation of the different schedules of health professions schools, and the amount of time and resources required to conduct IPE activities are common problems listed in the literature [5].

While there has been an influx of literature over the last decade on ways to implement IPE learning activities in academic environments [6–9], an extracurricular activity involving the utilization of a nonfiction book across various professions presents a new IPE learning opportunity. While book discussions have been used many times in health professions education to encourage professionalism [10], cultural competence [11–17], leadership [14–15, 18–21], empathy and compassion [13, 22–26], ethics [12, 27–29], spiritual care [30], and other outcomes [13, 19, 31–34], Kilham and Griffiths found no studies in the literature using a standalone book discussion activity for IPE purposes [35].

Books about patient or health care provider experiences allow readers to reflect on the often sensitive or shocking parts of the story in a safe environment when they are emotionally ready [14, 24]. Book discussions present issues in “an environment that encourages the students to share their insights and solutions to patient and professional dilemmas” [26]. This makes an IPE book discussion an ideal activity to allow students to engage with others from different disciplines, while also achieving the definition of IPE as outlined by the World Health Organization [1].

In the past, librarians have been a part of book discussions, but many times their participation has followed the traditional role of recommending books or teaching participants how to find resources [19, 31]. However, librarians can perform a wide variety of roles in planning educational activities. IPE book discussions provide an exciting opportunity for librarians to integrate into IPE initiatives and bring nontraditional skills relating to development of pedagogy, instructional design, and information organization and management. The inclusion of librarians as members of the IPE planning team can help solve traditional barriers to IPE activities such as lack of leadership, lack of enthusiasm, and the challenge of time and resources.

## STUDY PURPOSE

This paper outlines a specific IPE book discussion activity related to Paul Kalanithi’s *When Breath Becomes Air* [36] that included a total of eighty students and thirty-nine faculty representing all health affairs schools. The reasons for selection of this book, the creation of a discussion guide, and the process and implementation of twenty-nine small IPE discussions are described. Additionally, pre– and post–book discussion survey results and information on scheduling and communication strategies used with all participants before, during, and after the activity to assist with replication over time and across institutions are presented.

## CASE PRESENTATION

### Rationale for interprofessional education (IPE) activity

The IPE Steering Committee comprises faculty and staff representing all health professions schools (allied health, dentistry, medicine, nursing, pharmacy, public health, and social work) at our institution as well as health sciences librarians. Established in 2016, the IPE Steering Committee meets monthly to discuss existing IPE activities at the university and to strategize the implementation of new activities. While the IPE Steering Committee has had success with robust didactic and clinical activities, the committee needed to engage students who had not yet matriculated into their respective programs, as evidence demonstrates the importance of initiating IPE activities early in the curriculum.

Creating an IPE opportunity that coincides with the socialization of students to their professional schools encourages students to engage with other professions as well as members of their own profession. This opportunity was intended to help break down stereotypes or myths early in student learning as well give learners an opportunity to ask questions about other professions. The timing of the activity was purposefully planned for the beginning of the fall semester to best establish IPE as an institutional norm. Additionally, given the diverse role of various faculty and librarians from across the health affairs school, the IPE Steering Committee modeled shared leadership and enthusiasm for team work to the students who participated in this event.

Scheduling the activity was recognized as a potential challenge, as each respective school had a variety of material that needed to be covered during their respective orientation programs. To address this, the IPE Steering Committee needed to identify an activity that could be easily implemented and still enable rich discussion. Because these were incoming students, not all participants would have specific clinical skills, so the activity needed to allow participation from a professional lens without requiring in-depth health care knowledge [10, 29]. A book discussion was ideal because it enabled students who were just beginning their health programs to share their reactions to the book from their professional viewpoints as part of an interprofessional group.

This activity also served as an opportunity to highlight the skills and contributions of the health sciences librarians as part of an interprofessional health sciences education team: three librarians who volunteered on the IPE Steering Committee participated in all aspects of planning, conducting, and assessing the activity. The involvement of the librarians throughout this project has led to additional opportunities for them to share their expertise in health sciences information and education and to participate in IPE initiatives on campus.

### Activity planning

The IPE Steering Committee planned to implement the book discussion in August, so organization of the event began the previous March. Topics were brainstormed that could be applicable to all professions, and a focus on end-of-life care was identified as a subject matter that needed more coverage in the university’s curricula. The health sciences librarians and faculty generated a list of books on the topic with a summary, cost of the books, and availability. Using a survey developed by health sciences librarians, committee members then ranked their preferences, and the highest ranked book was selected.

### Book selection and purpose

*When Breath Becomes Air* explores the end-of-life experience of Kalanithi, a young neurosurgical resident who was unexpectedly diagnosed with terminal cancer [36, 37]. Students find IPE events most meaningful when they can be translated into current and future practice [38], and Kalanithi’s book prompted students to contemplate his journey from diagnosis to his passing, while reflecting from their own personal and professional perspectives. From an educational perspective, the book addresses team-based care, coordination, and personal, familial, and system perspectives of a major health diagnosis, making it a good fit for health sciences students.

### Implementation process

The IPE Steering Committee met twice a month from May to July to organize the book discussion. The committee also consulted with coordinators from an existing campus first-year reading program to build on their successful strategies for implementation. Next, each health profession faculty member contacted their individual school’s orientation director and distributed the book information and a brief overview of the activity as part of the orientation packet mailed to all incoming students in mid-May. Additionally, an email about the event was distributed to current students and faculty. Faculty from all health professions schools were notified of the upcoming book discussions and invited to participate as facilitators of small groups.

The health sciences librarians were involved with the project in several ways through developing the discussion framework and materials, assessing students’ learning, and coordinating administrative tasks, all of which expanded on the role of librarians in previous studies [19, 31, 34]. Two of the librarians developed the pre- and post-discussion surveys for students and group facilitators in Qualtrics, using their prior experience with online educational assessment. A librarian was also involved in administrative coordination of the activity, including reserving rooms, designing the initial facilitator and student availability surveys, and scheduling facilitators. The team sorted students into interprofessional groups based on their availability, working to maximize the diversity of disciplines represented in each session and broadly enable IPE to take place in each discussion group.

The IPE Steering Committee chose to hold as many book discussions as possible in the health sciences library for two reasons. Practically, the committee wanted to utilize the centrally located meeting spaces that were available in the health sciences library as a convenient, familiar place for students and faculty facilitators to hold their discussions. Philosophically, the committee wanted to convene these discussion groups in a central learning environment that was accessible to every health program on campus and was “program neutral” [34, 38]. For some discussion group sessions, when a library space was not available, the discussions were scheduled in various health schools on campus, rotating which buildings were utilized.

The IPE Steering Committee adapted a discussion guide from the School of Pharmacy and the book’s online reader’s guide [39]. This document was sent to the faculty facilitators shortly before the discussion sessions. The guide included the session’s learning objectives, primary discussion questions, and tips for facilitators to generate dialogue among the students. The supplemental appendix includes the discussion questions, which were adapted from Penguin Random House reader’s guide [39] to be relevant for students across different disciplines and to generate discussion around the key themes of the book. After each discussion group, faculty and student participants were sent thank you emails and a document containing IPE resources, thoughts about resilience, and a link to campus wellness resources and advice on providing end-of-life care, in case the discussions proved to be distressing.

### Participants

A total of eighty students and thirty-nine faculty, representing seven different health affairs schools, participated in the twenty-nine book discussions held in August and September of 2017. This activity took place over a one-hour period and occurred once. The activity covered the entire book, rather than split it into multiple sessions. One to two health affairs faculty or staff facilitated each discussion session with four to nine students. Students indicated their preferences for timing of the sessions, but they were assigned to groups based on their disciplines to create more diverse groups because previous studies indicate that self-selected groups have less rich conversations [35].

The student learners ranged from undergraduate nursing (BSN) students to master’s and doctoral students from all health affairs schools. Table 1 lists the percentage of students participating from each of the health professions schools. Table 2 lists the percentage of faculty members participating from each of the health professions schools.

**Table 1:** Breakdown of student participants by health professions school

Health professions school	Percentage of participants (%)	Total (n=80)
Nursing	16%	n=13
Public health	33%	n=26
Medicine	16%	n=13
Social work	8%	n=6
Dentistry	21%	n=17
Allied health	3%	n=2
Pharmacy	4%	n=3

**Table 2:** Breakdown of faculty participants by health professions school

Health professions school	Percentage of participants (%)	Total (n=39)
Nursing	13%	n=5
Public health	33%	n=13
Medicine	31%	n=12
Social work	8%	n=3
Dentistry	5%	n=2
Allied health	3%	n=1
Pharmacy	8%	n=3

### Pre- and post-survey results

Students were sent an email prior to the activity that included their scheduled book discussion time and room information, along with a scale to assess comfort levels regarding interprofessional roles and communication. Following the activity, students were sent another survey link with the same questions assessing their comfort with interprofessional roles and communication and evaluation questions that focused on their satisfaction with the activity. This evaluation was sent via Qualtrics approximately one week after the book discussion, and results from student post-surveys are presented in Table 3.

**Table 3:** Pre- and post-survey results for student interprofessional education (IPE) knowledge and attitudes (n=33)

		Strongly agree	Agree	Neither agree nor disagree	Disagree	Strongly disagree
Currently, I am comfortable with the idea of interprofessional team-based care.	Pre	45%	46%	80%	1%	—
	Post	73%	21%	3%	3%	—
I am comfortable describing my professional role to another team member.	Pre	25%	54%	16%	4%	1%
	Post	50%	38%	9%	5%	—
I am comfortable describing another professionals’ role.	Pre	10%	33%	43%	14%	1%
	Post	15%	55%	24%	6%	—
Interprofessional collaboration is essential when providing care for patients and their families.	Pre	75%	24%	1%	—	—
	Post	88%	12%	—	—	—
The aim of this book discussion was to provide an opportunity for students from multiple health professions to learn from, with, and about each other. To what extent do you agree or disagree that we achieved this goal?	Post	29%	62%	—	9%	—

Faculty and students were also asked questions in the post-survey to evaluate the format and implementation of the book club. Qualitative themes taken from open response post-survey questions found that students appreciated the unique perspectives, dialogues, and interprofessional connections that they made through this book discussion. Faculty valued the thoughtful contributions, interprofessional connections, and the quality of the book selected for this event. Faculty and students both felt the book discussion program could benefit from more interprofessional diversity and a higher number of participants. For both faculty and students, the greatest value of the book discussion was the ability to interact with other disciplines and to engage in collaborative discussion.

## DISCUSSION

### Lessons learned and next steps

Overall, the IPE Steering Committee found the book discussion pilot program to be successful based on anecdotal feedback and post-survey results, despite some last-minute challenges. It is important to plan enough time to think through the entire event from learning objectives to dissemination [39]. Because this project needed to be implemented in time for the beginning of the fall semester, committee members approached problems or needs as they arose in the planning process with little time for anticipation. The IPE Steering Committee could have benefited from additional time for organizing the event, planning the logistics, thinking through various issues, and delving more deeply into the desired student learning objectives of the event [40].

The biggest challenge was lack of time, both due to the steering committee being made up of faculty and staff volunteers and the tight deadline. Creating subcommittees or smaller teams to address each phase of planning, implementation, and wrap-up might facilitate a smoother organization process and save the steering committee time when planning future events.

A predetermined group of questions were sent to facilitators as an optional tool to guide discussions. Feedback shows some facilitators used the questions to an extent, whereas others did not use them at all. A missing piece of our project was a way to gather information about the nature of discussions, which would be helpful in informing project design and creating discussion questions and guides in the future. More information about the discussions might have also helped us understand survey responses from participants. It might also have been helpful to assign an IPE Steering Committee member the role of note-taker so that these observations could have been more systematically recorded and analyzed.

Clear and effective communication is an essential part of any project, especially one with many people and schedules to coordinate [38]. It is important to determine in advance what information will be needed, how often it will be needed, and how and with whom it will be shared. Facilitator feedback indicated that they want to better understand what the project goals were, why this project was chosen, whether it was mandatory for students, what participating students’ level of education was, and which health affairs schools were participating.

Organized by engaged faculty and staff on the IPE Steering Committee, this event was not an “orientation” activity mandated by any of the health professions schools or disciplines. Given that the home institution is the flagship university in the state, the participation numbers were low. However, in future years, efforts to more systematically coordinate this event to coincide with orientation activities and schedules could boost the participation rate. Future groups planning IPE book discussions may also want to consider alternate formats, such as book discussions through a learning management system or other web tools [41, 42].

The eighty participants and thirty-nine facilitators each received personal schedules and reminders via email, which was difficult for one person to manage alone and confusing for multiple people to manage together. Requests for faculty or student schedule changes were addressed as quickly as possible, and updated meeting schedules were emailed to groups. Because of the many schedule changes and the large variation in number of students from each discipline, some discussion groups, particularly toward the end of the project, were smaller than or not as diverse as originally intended. Utilizing an IPE group email address that several people can manage or having close communication between the event communicators could solve this problem in the future. Scheduling software might also assist with the many logistics involved in organizing the event [38].

Additionally, the low post-survey response rate might have been due to timing of the activity with the start-up of the semester. A better response rate could also have been achieved by administering the survey at the end of each session rather than via email later on.

Publicity and dissemination after the activity should also be considered during planning [38]. Photographs, a last-minute thought, were taken at a few groups and shared via Twitter and on the IPE Steering Committee website. Tweets with photos provided a way to keep the university’s health affairs community informed about the project and remind them about the IPE Steering Committee’s work. A report summarizing a broad overview of all IPE projects with results, including outcomes from this learning activity, should be shared widely within the university, even if not formally published. This communication would be particularly impactful since the IPE Steering Committee plans to organize more book discussions and will continue to need support from health affairs faculty, staff, and students.

Interprofessional book discussion groups as an introductory IPE activity have been well received by health affairs faculty and students at the university. The pilot program was informative in showing how much planning is needed for this type of learning activity, which will ensure a smoother process for future IPE book discussions. The health sciences librarians played an important role in the interprofessional planning process. The librarians’ in-depth, successful involvement with other members of the IPE Steering Committee throughout this project and the hosting of discussions in the library strengthened relationships between the health sciences library and the health affairs schools on campus. The IPE Steering Committee now formally includes three librarians as permanent members.

## SUPPLEMENTAL FILE

Appendix Guide for discussion facilitators
