# Correction

**DOI:** 10.5195/jmla.2019.800

**Published:** 2019-10-01

**VOLUME 107**

**107(3) July, page i, page 403, and page 410**

Haley J, Carlson McCall R, Zomorodi M, de Saxe Zerdan L, Beth Moreton B, Richardson L. Interprofessional collaboration between health sciences librarians and health professions faculty to implement a book club discussion for incoming students. J Med Libr Assoc. 2019 Jul;107(3):403–10. DOI: http://dx.doi.org/10.5195/jmla.2019.563.

Page i, page 403, and 410: Lisa de Saxe Zerden’s name is misspelled in the table of contents, author byline, and authors’ affiliations.

The table of contents entry should be:

Jen Haley, Rebecca Carlson McCall, Meg Zomorodi, Lisa de Saxe Zerden, Beth Moreton, Lee Richardson

The author byline should be:

**Jen Haley, MSN, RN, CNL; Rebecca Carlson McCall, MLS, AHIP; Meg Zomorodi, PhD, RN, CNL; Lisa de Saxe Zerden, PhD, MSW; Beth Moreton, MLS; Lee Richardson**

The author affiliation should be:

**Lisa de Saxe Zerden, PhD, MSW**, lzerden@email.unc.edu, Senior Associate Dean for Master’s in Social Work Education and Clinical Associate Professor, School of Social Work, University of North Carolina at Chapel Hill, Chapel Hill, NC

