# Supplementary material for: Interprofessional collaboration between health sciences librarians and health professions faculty to implement a book club discussion for incoming students
Source: J Med Libr Assoc. 2019 Jul 1;107(3):403–10. doi: 10.5195/jmla.2019.563 (PMC6579584; doi:10.5195/jmla.2019.563)
Supplement: Appendix [file jmla-107-403-s001.pdf]

JMLA Journal of the Medical Library Association

Supplemental content to
J Med Libr Assoc. Jul;107(3):dx.doi.org/10.5195/jmla.2019.563
www.jmla.mlanet.org
© Haley, McCall, Zomorodi, de Saxe Zerdan, Moreton, Richardson 2019

Interprofessional collaboration between health sciences librarians and health professions faculty to implement a book club discussion for incoming students: a case study

Jen Haley, MSN, RN, CNL; Rebecca Carlson McCall, MLS, AHIP; Meg Zomorodi, PhD, RN, CNL; Lisa de Saxe Zerdan, PhD, MSW; Beth Moreton, MLS, Lee Richardson

APPENDIX

Guide for discussion facilitators

Questions	Tips for discussion
1. Describe in one word how you felt after reading this book.	This is intended as an opening question to help get the students talking. Recommend everyone having a chance to express their thoughts before diving into deeper discussion but would also not draw it out too much for timing. Possible examples: upset, inspired, anxious, less afraid.
2. How did this book impact your thoughts about medical care? The patient-provider relationship? End-of-life care?	There were some good examples of the patient-provider relationship (Paul's father, Emma Hayward [his oncologist]) as well as some bad examples (overnight medical resident who omitted entering medications [Tarceva] and avoided calling his attending). Encourage discussing the differences between the two types.
3. What patient care experiences have you witnessed that may relate to what Paul experienced during his training and medical care?	If students do not want to talk about personal experiences, maybe focus in on media stories or friends/relatives' examples.
4. Is this a book you will continue thinking about, now that you are done? Do you find it having an impact on the way you go about your days or having a future impact as you start your curriculum?	[No tips provided for this question]
5. Throughout part I of the book, Paul describes a few instances of communicating with patients about their medical conditions. What were some significant interactions/stories that stood out to you?	Paul mentions multiple instances where he talked to patients at their level, engaged the family/caregivers in the discussion, worked with them through the shock of a life-changing diagnosis, and emphasized the significance of trust/relationship (he mentioned a time he observed his father talking with patients and the human connection). As future health care providers, it is important to make connections with our patients as it can lead to better health outcomes and serving as better health care providers.

JMLA Journal of the Medical Library Association

SC
Supplemental content to
J Med Libr Assoc. Jul;107(3):dx.doi.org/10.5195/jmla.2019.563
www.jmla.mlanet.org
© Haley, McCall, Zomorodi, de Saxe Zerdan, Moreton, Richardson 2019

Questions	Tips for discussion
6. How do you think the years Paul spent, tending to patients and training to be a neurosurgeon, affected the outlook he had on his own illness? When Paul wrote that the question he asked himself was not "why me," but "why not me," how did that strike you?	This question discussion can go a variety of ways, but some areas may involve the following:<br>– Paul's struggle with seeking to still be his "own physician" to his "patient self."<br>– How his training and talking with patients about their life-changing diagnoses now applied to himself and coming to terms that he's experiencing the same scenario.<br>– How his training and knowledge of medical conditions has set him up to be an active patient (advocate) in his own care.
7. Were there any other passages or sentences that struck you as particularly profound or moving?	Definitely worth speaking from the heart about as a facilitator. Some passages may include:<br>– Paul's statement: "My life had been building potential, potential that would now go unrealized."<br>– Paul's reflection on his friend Jeff who committed suicide when one of his patients died (health care provider suicide rates among trainees are high).<br>– Lucy's epilogue on Paul's experience.
